# Fine mapping and identification of the bright green leaf gene *BoBGL* in Chinese kale (*Brassica oleracea* var. alboglabra)

**DOI:** 10.3389/fpls.2024.1507968

**Published:** 2024-12-23

**Authors:** Qi Zhang, Chenchen Wang, Jialu Song, Liwei Gao, Wenjie Shen, Yi Liu, Daozong Chen, Chen Tan

**Affiliations:** College of Life Sciences, Gannan Normal University, Ganzhou, China

**Keywords:** bright green leaf, *Brassica oleracea*, Chinese kale, fine mapping, wax biosynthesis

## Abstract

**Introduction:**

Chinese kale (*Brassica oleracea* var. alboglabra), is an annual herb belonging to the Brassica genus of Cruciferae, and is one of the famous specialty vegetables of southern China. Some varieties show bright green leaf (BGL) traits and have better commerciality. However, the genes responsible for this trait remain unidentified.

**Methods:**

In this study, gene mapping was measured by BSR-Seq and molecular marker analysis. Gene expression analysis was performed qRT-PCR. Cloning and sequence analysis of candidate genes were also performed.

**Results:**

Genetic analysis revealed that the bright green leaf trait is a dominant trait governed by a single pair of genes. BSR-seq and molecular marker validation mapped the candidate interval to about 1.5 Mb on chromosome C8. After expanding the BC1 population and analyzing recombinant individuals, the interval was refined to approximately 102 kb on chromosome C8 (50,787,908- 50,890,279 bp). There are 24 genes in this region, and after annotation and expression analysis, *BolC8t52930H (BoCER1.C8)*, associated with wax synthesis, emerged as a key candidate for *BoBGL*. We cloned this gene from both parents, revealing significant differences in their promoter regions. A co-segregation primer was subsequently developed and validated in a segregated population, with results consistent with expectations.

**Discussion:**

The gene *BoCER1.C8* is a potential candidate for controlling the bright green leaf trait in Chinese kale, and its function needs to be validated next. Mapping and cloning this gene is crucial to understanding wax synthesis regulation and developing new bright green leafy varieties of Chinese kale.

## Introduction

Chinese kale (*Brassica oleracea* var. alboglabra, 2n=18), is an annual herb in the crucifer family. It is one of the famous specialty vegetables of South China. The main edible organs of Chinese kale are the tender, fleshy stalk and the tender leaves. The flesh is crisp, tender and sweet, rich in nutrients, and popular with consumers (Zeng et al., 2021; Zhao et al., 2021). Bright green Chinese kale, which features bright green and shiny leaves and stems, is a better-selling commodity than regular Chinese kale and is favored by consumers. However, the genes associated with the bright green leaf (BGL) properties of Chinese kale have not been mapped and cloned, since this an important trait, we would like to identify the genes controlling the trait, which will be useful for breeding.

Studies have shown that the appearance of bright green leaves is mostly caused by the reduction or loss of wax in the plant epidermis (Wang et al., 2023; Liu et al., 2017b). There have been significant recent advances in the study of wax-related genes and transport regulatory mechanisms in plants. Many genes involved in wax biosynthesis and metabolism have been cloned. These genes are involved in the three wax metabolism related processes of plant wax synthesis, transport processes, and regulatory pathways. The genes involved in wax biosynthesis include *CER1, CER2, CER3, CER4, CER6, CER8, CER10, KCS1, KCS2, KCR1/KCR2, LACS2, FATB, MAH1, WAX2*, etc (Lee and Suh, 2015, 2022). Genes such as *CER5/ABCG12*, *PEC1/ABCG32* and *WBC11* are involved in wax transport (McFarlane et al., 2010). Additionally, key regulatory genes like *WIN1/SHN*, *MYB16*, *MYB30*, *MYB94*, *MYB96*, *MYB106*, *WRINKLED4*, *DEWAX*, *WAR3* and *WAR4* have been highlighted in studies (Kannangara et al., 2007; Seo et al., 2011; Go et al., 2014; Bernard and Joubès, 2013; Lee and Suh, 2015, 2022).

In *Brassica* plants, most of the studies on wax synthesis and regulation focus on genetic analysis, and only a few wax-related genes are mapped or cloned. An example is *BnaA.GL*, a gene homologous to *CER1* in *Arabidopsis* that is responsible for wax deficiency in *B.napus*, located at the end of chromosome A9 (Pu et al., 2013). The wax genes were located on chromosome A1 or A9, and the candidate genes included *CER1* (Yang et al., 2022a), *CER2* (Zhang et al., 2013; Li et al., 2023) and *CER60* (Yang et al., 2022b). Fine mapping of some bright leaf genes in cabbage has also been reported. Candidate genes include *CER1* homologous genes (Liu et al., 2017b; Ji et al., 2018); *CER2* homologous genes (Han et al., 2021; Ji et al., 2021) and *CER4* homologous gene (Liu et al., 2017a). Therefore, in general, there are few wax-related genes currently mapped and cloned in *Brassica* crops, which need to be further explored.

In this study, the bright green leaf mutant material ‘BGL’ of Chinese kale was used as the research object, which was crossed with the wild-type material to obtain the F_1_ and BC_1_ segregating population. Through bulk segregant analysis RNA sequencing (BSR-seq) and fine mapping, we identified the genes responsible for the bright green leaf trait. The establishment of co-segregated InDel markers will be helpful for molecular marker-assisted breeding of leaf type at seedling stage. This basic work will help to further analyze the wax synthesis regulation pathway of Chinese kale and the cultivation of new varieties of bright green leaf Chinese kale is of great significance.

## Materials and methods

### Plant materials and bulking for bright green leaf trait

**Figure 1:**
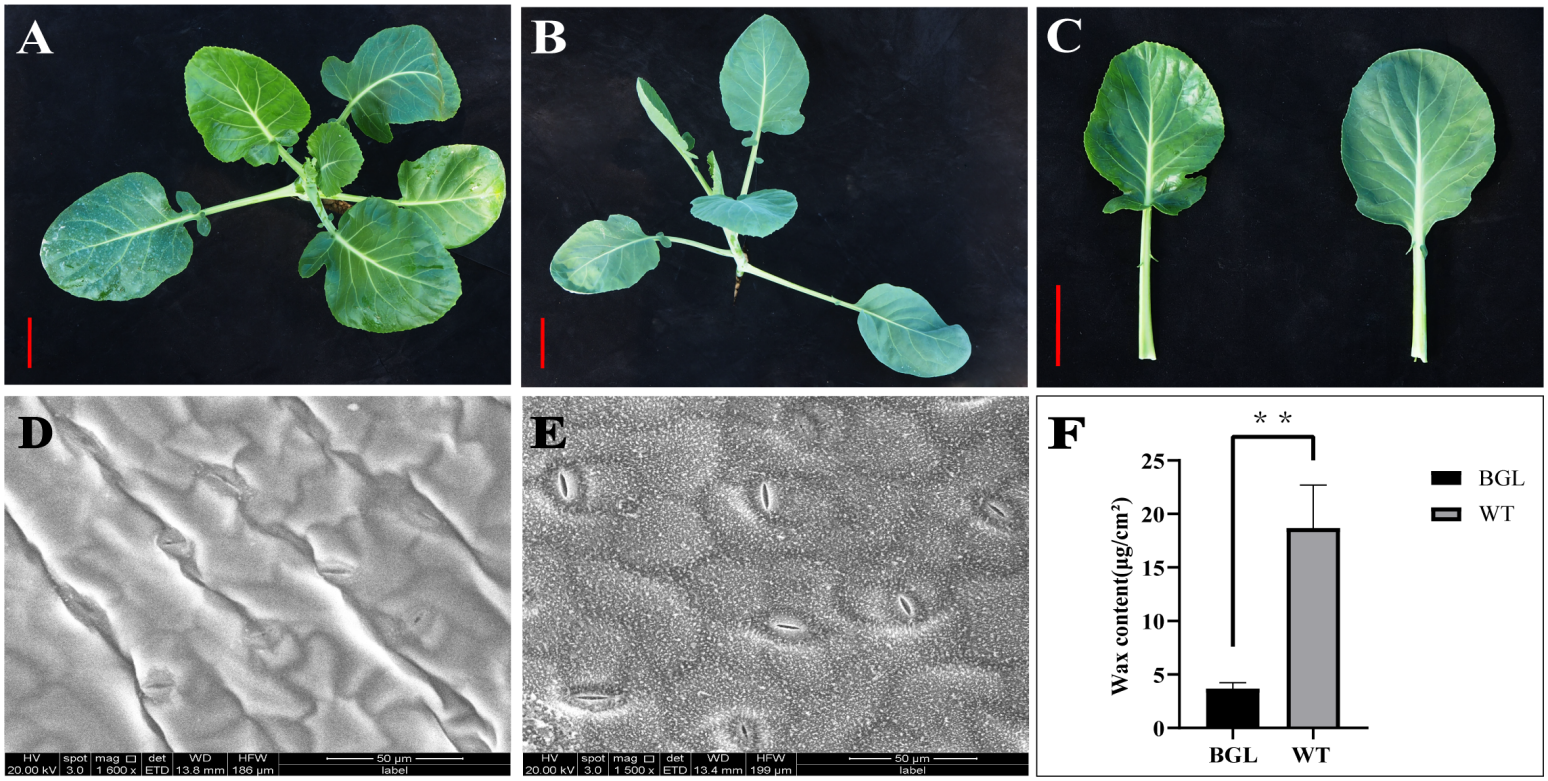
Phenotype and wax content of the studied materials. **(A)** Parent P1 (BGL); **(B)** Parent P2 (WT); **(C)** Comparison of leaf phenotypes between the two parents, scale = 5cm; **(D)** Appearance of leaf surface of BGL by SEM; **(E)** Appearance of leaf surface of WT by SEM; **(F)** Total wax content of BGL and WT, **Represents p <0.01.

The ‘BGL’ mutant (P1), characterized by its sterility and bright green leaves, was utilized as one parent, whereas the ‘WT’ parent (P2) exhibited fertility and a standard leaf phenotype (**Figure 1**). These two parental lines were crossed to produce the F_1_ hybrid generation (BGL×WT). Subsequently, Fifty F_1_ plants of bright green leaf type were used to obtain BC_1_F_1_ (F_1_×WT) generation. Leaf phenotypes of the F_1_ and BC_1_F_1_ generations were observed and recorded at the 7-leaf seedling stage. The bright green leaf bulk (Bulk-A) and the ordinary leaf bulk (Bulk-B) were selected from the BC_1_F_1_ generation based on leaf phenotype. An equal quantity of young leaves was promptly harvested, immediately placed in liquid nitrogen, and stored at -80°C for subsequent RNA extraction. The experimental materials were cultivated in a basic greenhouse at Gannan Normal University (N25°47′, E114°52′).

### RNA extraction and sequencing

Total RNA was extracted utilizing the Trizol Reagent (Invitrogen Life Technologies) and subsequently quantified using both the NanoDrop spectrophotometer (Thermo Scientific) and the Qubit 4.0 fluorometer. Quality control was conducted employing the Agilent 2100/4200 system (Agilent Technologies). Equivalent amounts of purified RNA from each of the 30 samples in Bulk-A and Bulk-B were pooled for RNA library construction. The libraries were constructed following the manufacturer’s protocols, and sequencing was carried out on the HiSeq 4000 platform. Raw sequencing reads were subjected to quality control using Fastp software (version 0.19.7). Clean reads were subsequently aligned to the *B. oleracea* reference genome (Braol_HDEM_V1.0) using BWA software. The BoBGL locus was mapped using MMAPPR software (Hill et al., 2013), briefly, Bam files were processed with the mpileup tool from the SAMtools package to generate a pileup file, which organizes the data into a position-based format displaying sequenced bases at each location. Reads were filtered based on specified minimum base and mapping quality thresholds, and allele frequencies were calculated. The resulting data were then analyzed in R for signal processing and peak identification. R plots the Loess fits and lists SNPs enriched in the mutant pool (allele frequency >0.75, Euclidean distance >0.5). These SNPs are filtered for nonsynonymous variants using Alleler and gene annotations, with results exported to a file. MMAPPR uses these optimized default values. The association analysis was conducted using the Δ(SNP-index) method with the identified SNPs and InDels.

### Determination of wax content

Mature leaves were utilized for the quantification of wax content. Leaves of appropriate size were immersed in 15 mL of chloroform for 30 seconds to extract the total epidermal wax mixture. Subsequently, 20 μg of n-tetracosane (C24) was added as an internal standard. The extract was then dried under a nitrogen (N_2_) stream to a volume of 1 mL and transferred to a pre-weighed and recorded gas chromatography (GC) vial. Following the evaporation of the remaining liquid under nitrogen, 100 μL of trimethylsilyl reagent was added, and the sample was derivatized in a 70°C incubator for 60 minutes. All wax samples were converted into trimethylsilyl derivatives, subsequently dried using nitrogen gas, and then weighed. The total quantity of cuticular wax was normalized to unit leaf surface area. The experiment was conducted with three biological replicates.

### Scanning electron microscopy analysis

Fresh mature leaf tissue was collected and fixed using a 2.5% glutaraldehyde solution for 2-4 hours. Subsequently, the samples were rinsed three times with 0.1 M phosphate buffer (pH 7.0) and then treated with a 1% glutaric acid solution in 0.1 M phosphate buffer (pH 7.0) for 1-3 hours. This was followed by three additional rinses with 0.1 M phosphate buffer (pH 7.0). The samples were then subjected to a dehydration series using ethanol concentrations of 50%, 70%, 80%, 90%, 95%, and 100% (twice) for 15 minutes each. After dehydration, the samples were soaked in a 1:1 mixture of 100% ethanol and isoamyl acetate for 30 minutes, followed by an overnight soak in pure isoamyl acetate. The samples were subsequently dried and transferred to a preparative chamber for vacuum coating. The samples were photographed using a scanning electron microscope (SEM) system (FEI Quanta 450).

### DNA extraction and molecular marker analysis

Genomic DNA was extracted from young leaves using the CTAB method and diluted to 50 ng/µL with 1× TE solution. The InDels in the candidate interval from BSR-seq were used to design markers to narrow down the BoBGL locus. Primers were designed utilizing Primer3 software, the sequences corresponding to the candidate intervals were extracted, with the product size specified to range from 180 to 350 bp, allowing for one mismatched base. Primers were synthesized by Wuhan Tianyi Huayu Gene Technology Co., Ltd (Wuhan, China). The 10 µL PCR mixture included 2 µL of template DNA (50 ng/µL), 5 µL of 2× FineTaq^®^ PCR SuperMix, 0.5 µL of each primer (10 µM), and 2 µL of sterilized ddH_2_O. The PCR program was: 94°C for 5min; 9 cycles of 94°C for 30s, 60°C for 30s (decreasing by 0.5°C per cycle), 72°C for 30s; 30 cycles of 94°C for 30s, 55°C for 30s, 72°C for 30s; and a final elongation at 72°C for 10min. PCR products were then separated on 6% polyacrylamide gel electrophoresis (PAGE).

### Identification and cloning of candidate genes

The DNA of 940 BC_1_F_1_ recessive plants was subjected to amplification using polymorphic primers, followed by the screening of recombinant plants. Subsequently, the genetic distance between the markers was determined, and a genetic map was constructed. Two markers proximal to the target site were identified. A physical map was then developed by utilizing the physical locations of these two markers on the *B. oleracea* reference genome (Braol_HDEM_V1.0) to delineate the candidate interval. Genes in the target intervals were annotated, and key ones were analyzed for expression via qRT-PCR. Specific primers for the *BoBGL* gene were used to amplify sequences from two parental lines, BGL and WT, using Primer5. The PCR products were cloned into the pMDTM19-T vector and transformed into DH5α cells. The candidate gene was then sequenced using the Sanger method. Subsequently, DNA sequencing reactions were conducted by Wuhan Tianyi Huayu Gene Technology Co., Ltd (Wuhan, China). The primer sequences utilized for the molecular markers in this study are detailed in **Supplementary Table S1**.

## Results

### A dominant gene controls the production of bright green leaf traits in this study

P1 is a plant with bright green leaves (**Figure 1A**), and P2 is a plant with ordinary leaves (**Figure 1B**), and the wax content is significantly reduced in P2 (**Figure 1F**). The leaf surface of the hybrid F_1_ plants is characterized by bright green leaves. For the BC_1_F_1_ (F_1_×WT) offspring, the number of ordinary leaves and bright green leaves is 1:1. Furthermore, the number of common leaves and bright green leaves in BC_2_F_1_ was also 1:1 (**Table 1**). It can be judged that the bright green leaf trait of cabbage is completely apparent inheritance controlled by a pair of alleles.

**Table 1:** Genetic studies were conducted on the bright green leaf plants within various segregating populations.

Population name	Plants tested	Ordinary leaf plants	Bright green leaf plants	Mendelian expectations	χ² value
P_1_	15	-	15	-	-
P_2_	15	15	-	-	-
F_1_	50	-	50	-	-
BC_1_F_1_	369	177	192	1:1	0.61
BC_2_F_1_	2148	1105	1043	1:1	1.79

χ2<χ0.05 (3.84) is considered as significant

### The *BoBGL* gene is located on chromosome C8

**Figure 2:**
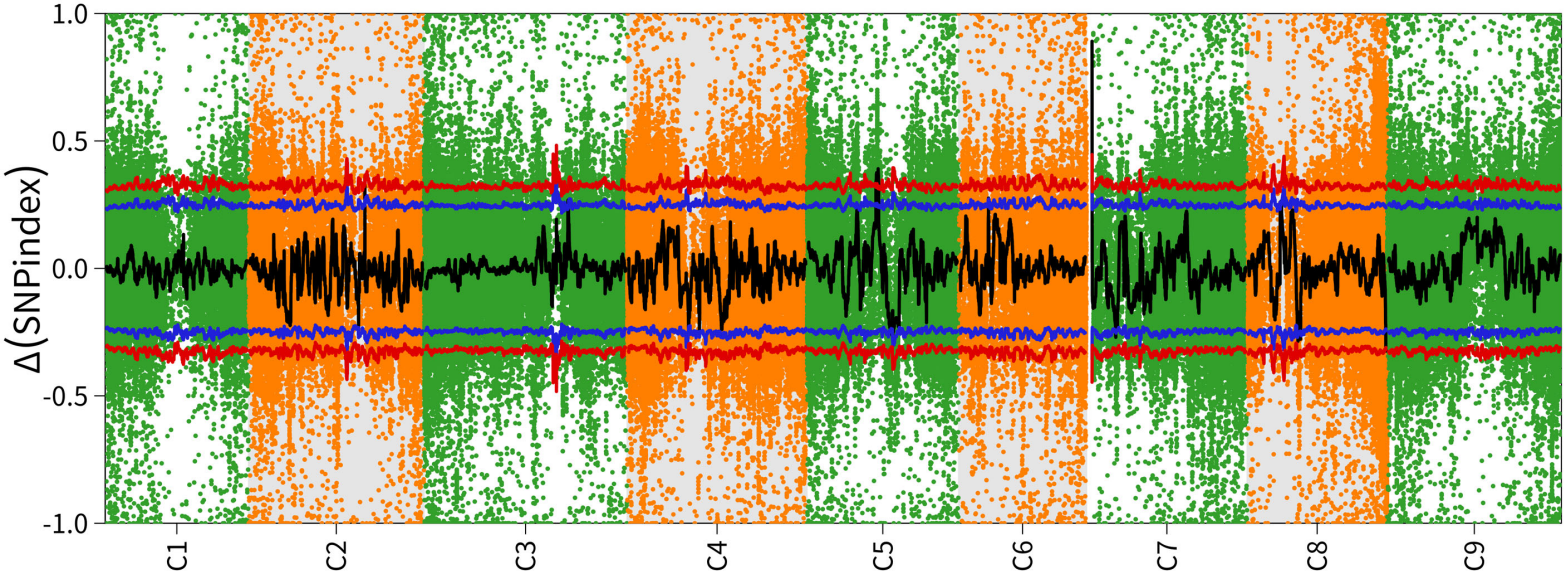
The chromosome-wise distribution of ΔSNP index values. Chromosome names (C1-C8) on the x-axis and colored points representing each SNP locus’s ΔSNP-index value. The black line shows the fitted ΔSNP-index value, and the red line marks the significance threshold.

The *BoBGL* locus was mapped using a mutation mapping pipeline for pooled RNA-seq (Hill et al., 2013). A total of 122,472,880 and 223,777,954 clean reads produced from the Bulk-A and Bulk-B RNA samples were mapped to the B. oleracea reference genome (Braol_HDEM_V1.0/, http://brassicadb.cn/#/Download/), respectively (**Supplementary Table S1**). Over 94% of the reads were mapped to unigenes, resulting in the discovery of 247,090 SNPs and 68,667 InDels between the two bulks. SNP-index was calculated according to the SNP of two progeny pools obtained by sequencing, which was used to map the gene of bright green leaf trait in Chinese kale. The Δ(SNP-index) of the two mixed pools was analyzed. The results showed that when the confidence level was 99%, the three regions C5, C7 and C8 distributed on the chromosome showed extremely significant peaks above the critical value level (**Figure 2**). The significant correlation intervals are: C5 (24.51-26.47Mb, ~1.96Mb), C7 (1.45-2.45Mb, ~1.00Mb), and C8 (49.36-51.42Mb, ~2.06Mb) (**Table 2**).

**Table 2:** Candidate genomic regions identified by the BSR-Seq analysis.

Chromosome ID	Start (bp)	End (bp)	Size (Mb)
C5	24510001	26470000	1.96
C7	1450001	2450000	1.00
C8	49360001	51421300	2.06

**Figure 3:**
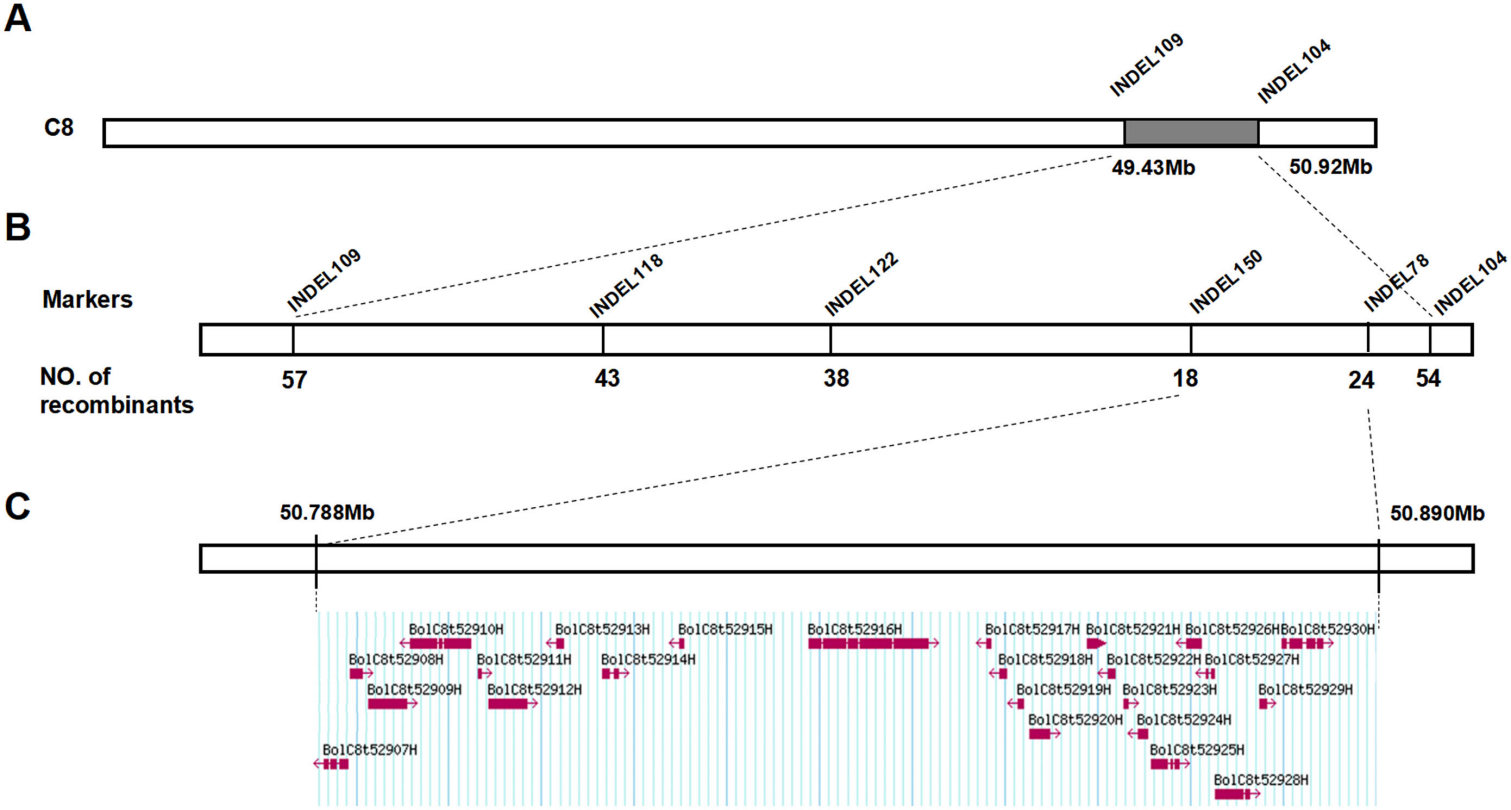
Mapping of the candidate gene controlling the bright green leaf trait. **(A)** BoBGL was initially located in the range of 49.88-50.92 Mb. **(B)** The positioning interval for the candidate region was narrowed using three InDel markers, and the flanking markers were Indel150 and Indel78. **(C)** The candidate interval contained 24 genes.

Subsequently, we used the molecular marker method to further verify the candidate interval. Firstly, we examined the phenotypes (bright green leaves and ordinary leaves) of a BC_1_ segregating population containing 186 individuals. Secondly, we developed some InDel markers covering all candidate intervals. Genotyping of the 186 BC_1_ segregating plants was conducted with 9 pairs of polymorphic InDel markers (**Supplementary Table S2**), resulting in the creation of a genetic linkage map. The findings indicated that the gene responsible for the bright green leaf gene, *BoGBL*, was situated on chromosome C8 between markers INDEL109 and INDEL104 (**Supplementary Figure S1**), with positions on the reference genome at 49.43 Mb and 50.92 Mb, as shown in **Figure 3A**. Therefore, the bright green leaf gene *BoGBL* can be preliminarily located in about 1.5 Mb interval between 49.43Mb and 50.92Mb on chromosome C8 of *B.oleracea*.

### Fine-mapping of the *BoBGL* gene

In order to further narrow the candidate interval, combined with the results of preliminary mapping, 940 BC1 populations (all ordinary leaf type) were screened for recombinant plants based on flanking markers (INDEL109, INDEL104), and a total of 81 recombinant plants were obtained. At the same time, four polymorphic InDel markers were used to genotype 81 recombinant plants and finely map the bright green leaf gene *BoBGL* in *B.oleracea*. The *BoBGL* locus was more precisely identified to be within a 102-kb region on chromosome C8 of the *B. oleracea* reference genome, located between the markers INDEL150 (50 787 908bp) and INDEL78 (50 890 279bp) (**Figure 3B**; **Supplementary Figure S1**).

### Identification of the candidate gene

**Figure 4:**
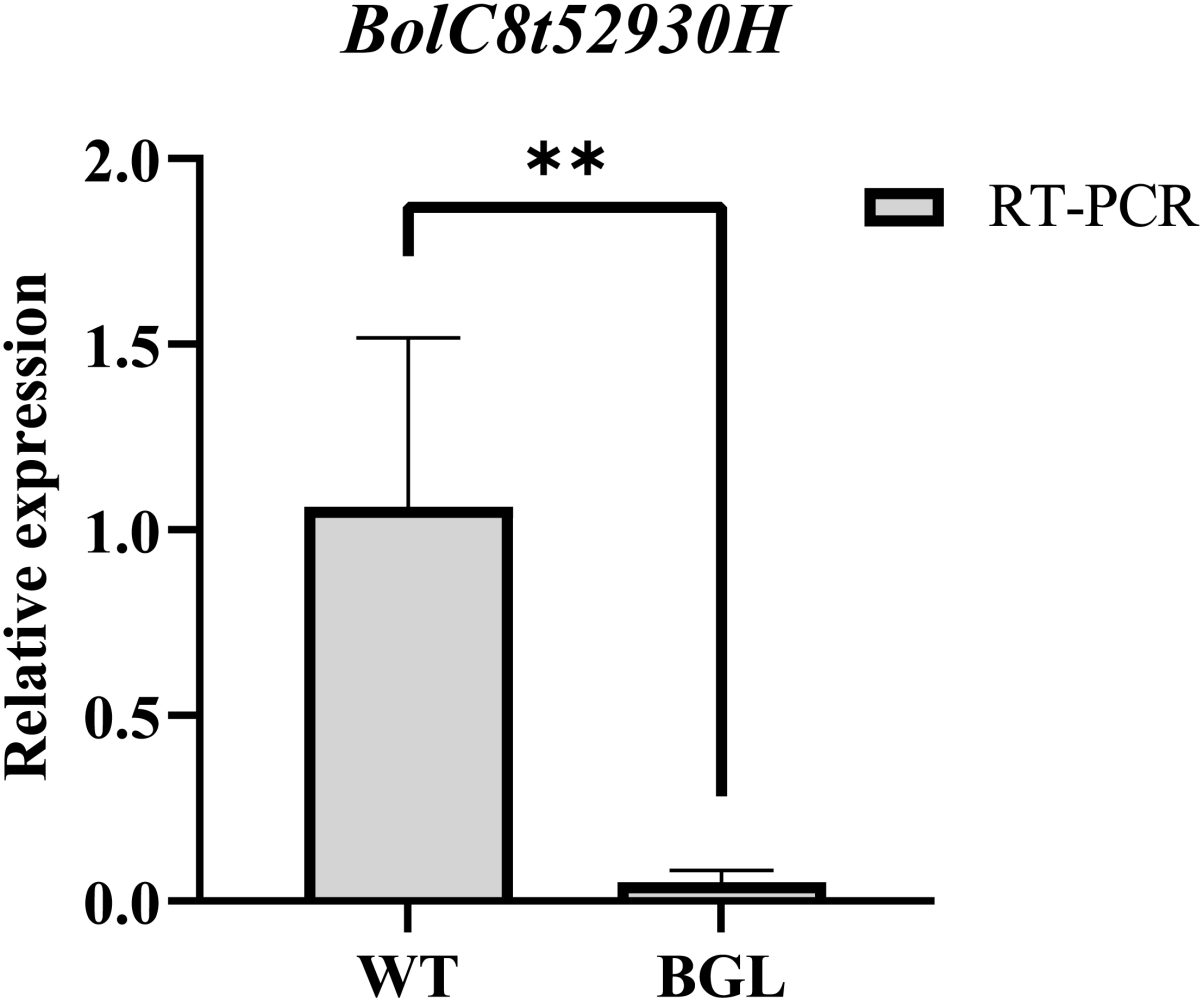
qRT-PCR validation of candidate gene. *BolC8t52930H* expression levels determined by qRT-PCR between BGL and WT. BoActin is used as an equal loading control. Error bars indicate standard errors from three biological replicates. ** denotes significant differences (p<0.01).

According to the location of the *BoBGL* locus, there were 24 genes within these 102-kb region in the reference *B. oleracea* genome (**Figure 3C**). To identify the key candidates, we conducted gene annotation on 24 genes contained in this region. The results showed that among the 24 genes, only the candidate gene *BolC8t52930H*, named as *BoCER1.C8*, was related to the biosynthesis of plant cuticle wax (**Table 3**). The gene is highly homologous to the *Arabidopsis* wax synthesis-related gene *AtCER1* (**Table 3**). In addition, qRT-PCR showed the gene *BolC8t52930H* was significantly differentially expressed in the two parents (**Figure 4**). In summary, the gene *BolC8t52930H* was identified as a key candidate gene for *BoBGL*.

**Table 3:** The annotated genes in the candidate genomic regions associated with the bright green leaf trait.

NO.	Gene ID	Position (bp)	Function annotation	Arabidopsis thaliana	Gene name
1	*BolC8t52907H*	C8: 50786567-50789198	Zinc finger protein-like protein	*AT1G01930*	–
2	*BolC8t52908H*	C8: 50789389-50790644	Cyclophilin-like peptidyl-prolyl cis-trans isomerase family protein	*AT1G01940*	*CYP18-1*
3	*BolC8t52909H*	C8: 50791272-50795437	Armadillo repeat kinesin 2	*AT1G01950*	*ARK2*
4	*BolC8t52910H*	C8: 50795900-50802501	SEC7-like guanine nucleotide exchange Family protein	*AT3G43300*	*MIN7*
5	*BolC8t52911H*	C8: 50803247-50803473	–	–	–
6	*BolC8t52912H*	C8: 50804262-50808395	Secretory 1A	*AT1G02010*	*SEC1A*
7	*BolC8t52913H*	C8: 50811598-50812374	C2H2-type zinc Finger Family protein	*AT4G04404*, *AT1G02040*	–
8	*BolC8t52914H*	C8: 50816537-50818245	Squamosa promoter binding protein-like 8	*AT1G02065*	*SPL8*
9	*BolC8t52915H*	C8: 50824869-50825378	Zinc ion-binding protein	*AT1G02070*	–
10	*BolC8t52916H*	C8: 50838842-50851729	Transcription regulator	*AT1G02080*	*NOT1*
11	*BolC8t52917H*	C8: 50858004-50858435	–	–	–
12	*BolC8t52918H*	C8: 50859445-50860089	–	–	–
13	*BolC8t52919H*	C8: 50861440-50862066	–	–	–
14	*BolC8t52920H*	C8: 50862682-50864763	Nucleic acid-binding%2C OB-Fold-like protein	*AT1G14800*	–
15	*BolC8t52921H*	C8: 50868921-50870786	PIF1 helicase	*AT5G28780*	–
16	*BolC8t52922H*	C8: 50871180-50871800	–	–	–
17	*BolC8t52923H*	C8: 50872749-50873249	Ethylene-responsive element binding Factor 13	*AT2G44840*	*ERF13*
18	*BolC8t52924H*	C8: 50874328-50875521	Proteasome component (PCI) domain protein	*AT1G02090*	*CSN7*
19	*BolC8t52925H*	C8: 50875833-50878712	Leucine carboxyl methyltransferase	*AT1G02100*	*LCMT1*
20	*BolC8t52926H*	C8: 50879522-50881195	RAS 5	*AT1G02130*	*RA-5*
21	*BolC8t52927H*	C8: 50881667-50882474	Mago nashi Family protein	*AT1G02140*	*MEE63*
22	*BolC8t52928H*	C8: 50882684-50886297	Homolog of asparagine-linked glycosylation 12	*AT1G02145*	*ALG12*
23	*BolC8t52929H*	C8: 50887446-50888010	–	–	–
24	*BolC8t52930H*	C8: 50889892-50894227	Fatty acid hydroxylase super Family	*AT1G02205*	*CER1*

### Gene cloning of BolC8t52930H

**Figure 5:**
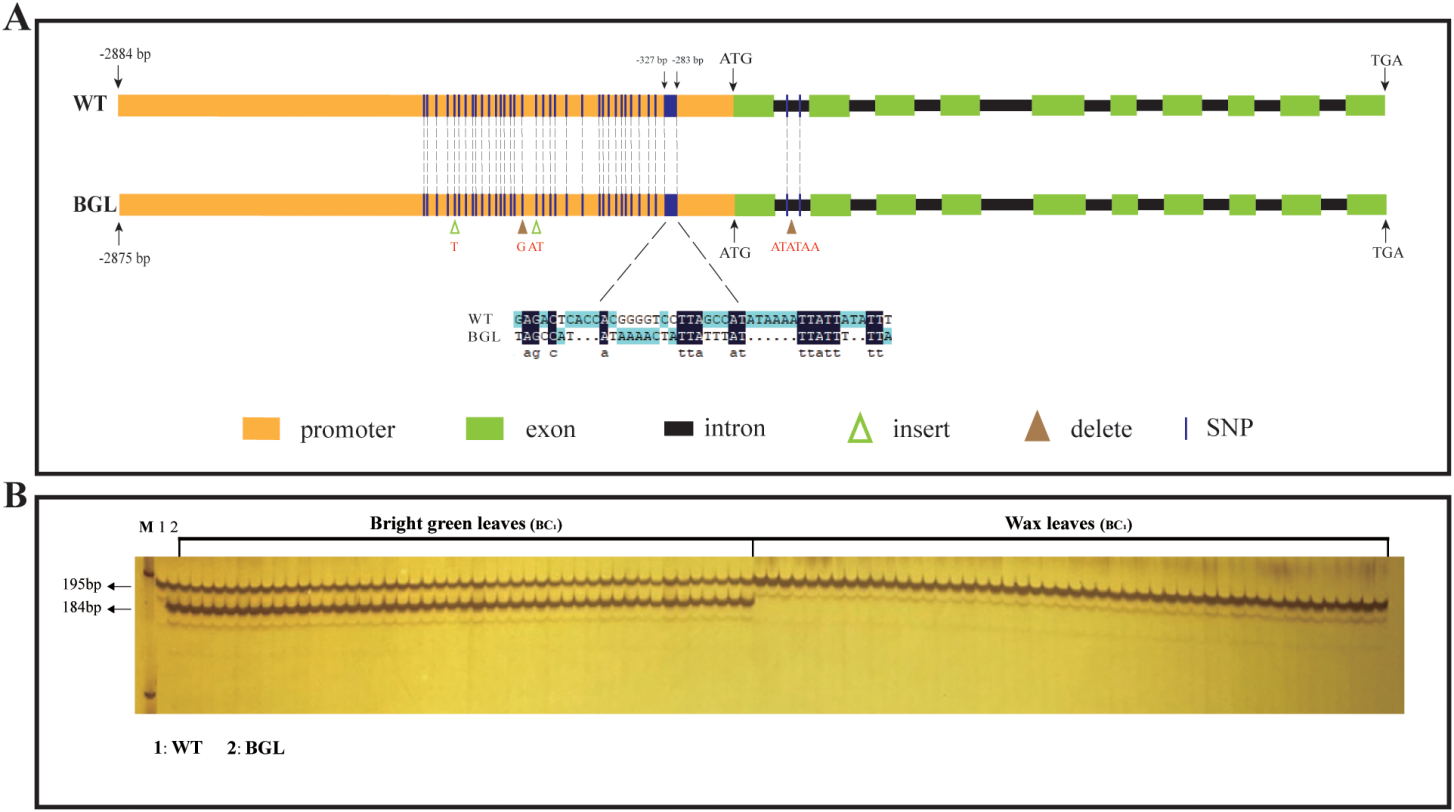
The structure of *BolC8t52930H* in the two parents and verification of co-segregation in BC_1_ generation. **(A)** Gene structure of *BolC8t52930H* and its mutation in BGL. Yellow boxes represent promoters, green boxes represent exons, black boxes between green boxes represent introns, green triangles indicate insert mutations, brown triangles indicate delete mutations, and dashed lines indicate SNP mutations. **(B)** The developed polymorphic InDel marker was used to analyze the co-segregation of BC_1_ genotypes.

To confirm our speculation, we cloned the genomic sequence and coding sequence of *BolC8t52930H* from two parental plants. The findings indicated that the coding sequences in the parental organisms were completely consistent, so as the protein sequence, despite minor variations in their genomic sequences (**Figure 5A**; **Supplementary Figure S2**). We also cloned the promoter sequence of *BolC8t52930H* from about 3K before the onset codon from both parents. There are some differences between the two promoters in the range of -280bp to -1500bp, with the largest being -282bp to -325bp (**Figure 5A**). The interval of about 40bp is significantly different from the insertion and deletion, and the differences in other intervals are mainly SNPs (about 30 ones), and 4 gaps (**Figure 5A**; **Supplementary Figure S2**). In summary, there are significant sequence differences in promoter regions between the two parents, and therefore, we speculate that variations in the promoter region of *BolC8t52930H* may be responsible for the changes in gene expression in bright green leaf material.

### The development of molecular markers for identifying bright green leaf genotypes

Next, we developed an polymorphic InDel marker using the sequence differences in the interval (-282bp to -325bp) with the largest difference in gene regions between parents to analyze whether *BoBGL* is co-segregated with bright green leaf traits. As we expected, the co-segregation analysis of 184 BC_1_ plants showed that their genotypes were completely co-segregated with their phenotypes, that is, bright green leaf individuals can amplify a 184bp fragment, while ordinary leaf individuals cannot (**Figure 5B**). Therefore, we developed a useful functional marker that can be used for marker-assisted selection of bright green leaf traits in *B. oleracea*.

## Discussion

Bright green leaf Chinese kale has shiny leaves and stems that are more commercially viable than ordinary Chinese kale. This study revealed a monogenic inheritance pattern for the bright green leaf trait in Chinese kale. Through BSR-seq and fine mapping, we mapped the gene for the bright green leaf trait of Chinese kale to a 102-kb region of 50 787 908- 50 890 279 bp on the C8 chromosome of *B.oleracea*. There are 24 genes annotated in this region, of which only *BolC8t52930H* is related to wax synthesis. It is a homologous gene of *AtCER1* in *A.thaliana*, so it is named *BoCER1.C8*. Gene expression analysis also confirmed that it showed strong differential expression between the two parents. Cloning *BoCER1.C8* revealed notable variations in sequence between the parental strains in the promoter regions. Finally, we developed an InDel marker and used it to verify the co-segregation of *BoCER1.C8* with bright green leaf traits. Finally, we concluded that *BoCER1.C8* is the gene corresponding to the bright green leaf trait in Chinese kale.

Due to the quick advancement of high-throughput sequencing technology, BSR-seq has the potential to serve as a valuable resource for initial gene mapping. BSR-seq is a commonly utilized technique in various crops for quickly and cost-effectively mapping genes of interest in plants. For example, wheat (Shi et al., 2021; Wang et al., 2022), soybean (Huang et al., 2024), Sugarcane (Cheng et al., 2022; Wu et al., 2022), the Black Lemma (Liu et al., 2023), foxtail millet (Tian et al., 2022), etc. In this study, we used BSR-seq to quickly lock the gene *BoBGL*, which controls the bright leaf trait, in the interval of about 1.5-Mb on the C8 chromosome, and obtained a large number of inter-parental SNP and InDel variations for marker development. This strategy greatly improves the efficiency of locating genes of interest.

The generation of bright leaf traits is usually caused by the loss or reduction of wax. In *Brassica* plants, some bright color trait genes have been mapped or cloned, which are basically related to wax synthesis and regulation (Pu et al., 2013; Zhang et al., 2013; Liu et al., 2017c, 2017; Ji et al., 2021; Han et al., 2021; Yang et al., 2022b; Li et al., 2023; Wang et al., 2023). Several genes associated with bright leaf characteristics in *B.oleracea* have been identified through mapping or cloning, such as *Bol018504* (*CER1*) (Liu et al., 2017b, 2017c; Ji et al., 2018), *Bo1g039030* (*BoCER2*) (Ji et al., 2021; Han et al., 2021), *Bol013612* (*CER4*) (Liu et al., 2017a), and *Bol026949* (Wang et al., 2023). In the current study, the primary candidate gene was identified on the *CER1* gene located on the C8 chromosome, a finding that aligns with several previous studies (Liu et al., 2017b, 2017c; Ji et al., 2018). However, our research proposes that the bright leaf trait in Chinese kale is regulated by a dominant gene, a contrast to other reports suggesting control by a recessive gene (Liu et al., 2017b, 2017c; Ji et al., 2018). Notably, a 2,722-bp insertion in the first intron of *Bol018504* (*CER1*) in the glossy mutant was found to result in an alteration in the RNA splice site (Liu et al., 2017c). Furthermore, the fourth intron of *BoCER1* in the glossy mutant was observed to incorporate a 252-bp insertion (Ji et al., 2018). In this study, we found some base variations in the promoter region, which may lead to dominant inheritance, but the specific mechanism is not clear. Dominance is a genetic interaction between alleles at a locus, often included in gene expression models based on molecular networks. It can occur at various integration levels, from allele-specific expression biases to organismal traits, involving diverse molecular interactions and physiological and developmental characteristics (Billiard et al., 2021). For this study, a possible mechanism is the trans-action of sRNA encoded by the flanking region of the dominant allele to induce transcriptional silencing of the recessive allele (Tarutani et al., 2010).

The gene *CER1* is responsible for producing an enzyme that creates alkanes, potentially interacting with *CER3* and various cytochrome b5 (CYTB5s) isoforms to facilitate the production of extremely long alkanes (Bernard et al., 2012; Pascal et al., 2019; Bourdenx et al., 2011). In *Arabidopsis*, the overexpression of the *CER1* gene has been shown to enhance the biosynthesis of very long-chain alkanes (Bourdenx et al., 2011). The *cer1-1* mutant, on the other hand, is distinguished by a pronounced reduction in the products of the alkane formation pathway, including alkanes, secondary alcohols, and ketones, with a concurrent increase in the corresponding aldehydes (Aarts et al., 1995). Furthermore, the role of *CER1* in the synthesis of long-chain alkanes has been substantiated in other plant species as well, such as cucumber (Wang et al., 2015), rice (Ni et al., 2018), Brachypodium distachyon (Wu et al., 2019), and tomato (Wu et al., 2022). In *Brassica* species, *CER1* was also proposed as the candidate gene for the glassy trait (Pu et al., 2013; Liu et al., 2017b, 2017c; Ji et al., 2018; Yang et al., 2022a). Specifically, in *B.napus*, the gene *BnaA.GL* has been precisely mapped to chromosome A09, with *BnCER1* identified as the candidate gene (Pu et al., 2013). Furthermore, in *B.rapa*, the cuticular wax biosynthesis gene, *BrWax2*, was identified through map-based cloning, proposing *Bra032670* (*CER1*) as the candidate gene (Yang et al., 2022a). In this study, we narrowed the interval to approximately 102-kb by fine mapping, including 24 candidate genes, including the *CER1* gene. The level of expression of the *CER1* gene was significantly reduced in the bright green leaf mutant. Sequence analysis also revealed a distinct sequence variation in the promoter region of the bright green leaf mutant, which may be responsible for the down-regulation of *CER1* gene expression. Therefore, we believe that the *CER1* gene is the target gene for the current status of the bright leaves of Chinese kale.

## Conclusion

In conclusion, we identified a candidate gene, *BolC8t52930H* (*BoCER1.C8*), associated with the bright green leaf trait in *Brassica oleracea* on chromosome C8. This identification was achieved using a genetic linkage map constructed through the re-sequencing of a BC_1_ segregating population derived from hybridization and backcrossing with the wild type (WT). We subsequently cloned this gene from both parental lines, uncovering significant differences in their promoter regions. A co-segregation marker was then developed and validated within a segregating population, successfully yielding the anticipated results. Consequently, the gene *BoCER1.C8* emerges as a potential candidate for regulating the bright green leaf trait in Chinese kale.

## Data Availability

The original contributions presented in the study are included in the article/**Supplementary Material**. Further inquiries can be directed to the corresponding author.

## Supplementary material

Supplementary Figure 1 Genetic linkage maps. **(A)** Preliminary mapping of genetic linkage map. **(B)** Fine mapping genetic linkage map.

Supplementary Figure 2 Sequence alignment analysis of candidate gene *BoCER1.C8*. **(A)** Amino acid sequence alignment diagram of coding region between the two parents. **(B)** Promoter sequence alignment between the two parents.
