# Supplementary figures and images for: Fine mapping and identification of the bright green leaf gene BoBGL in Chinese kale (Brassica oleracea var. alboglabra)
Source: Front Plant Sci. 2024 Dec 23;15:1507968. doi: 10.3389/fpls.2024.1507968 (PMC11701064; doi:10.3389/fpls.2024.1507968)

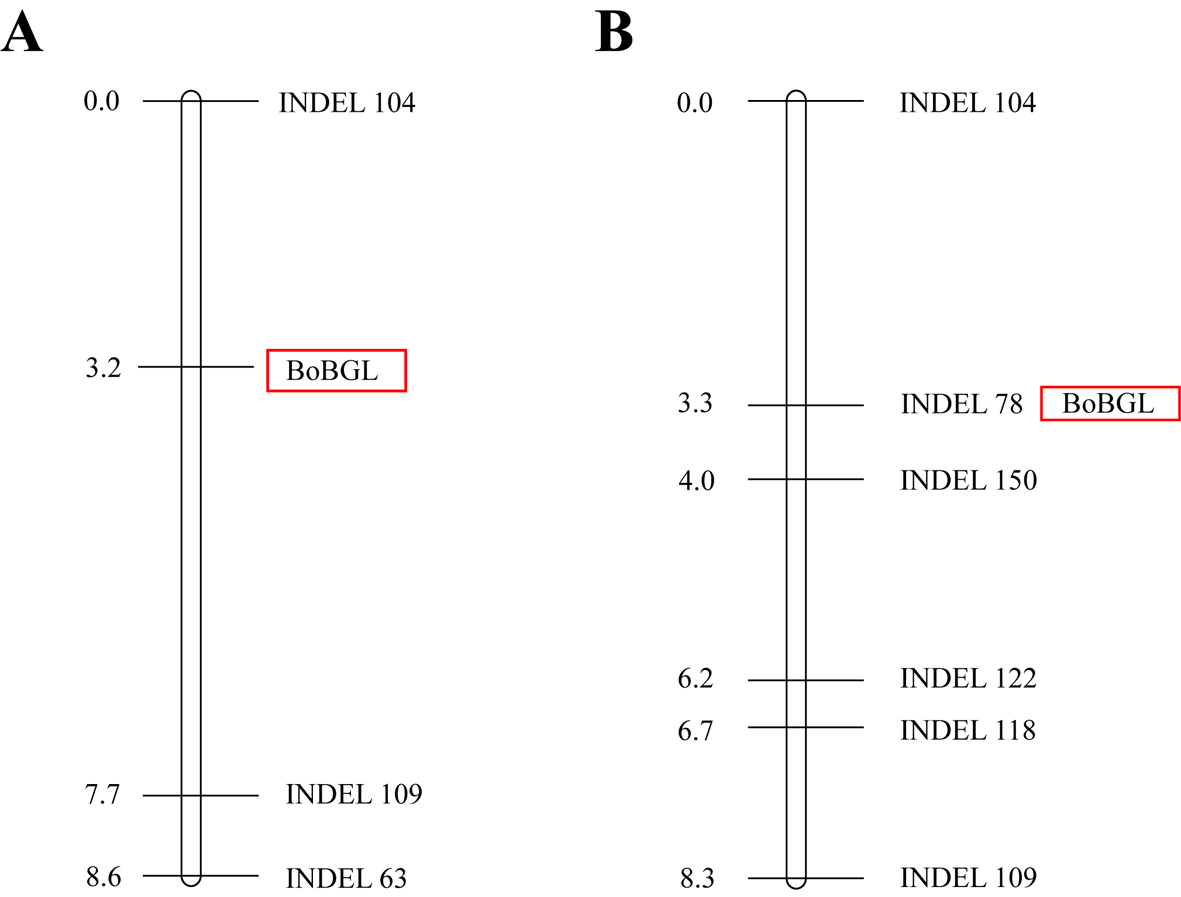

Supplement: Supplementary Figure 1 — Genetic linkage maps. (A) Preliminary mapping of genetic linkage map. (B) Fine mapping genetic linkage map. [file Image1.jpeg]

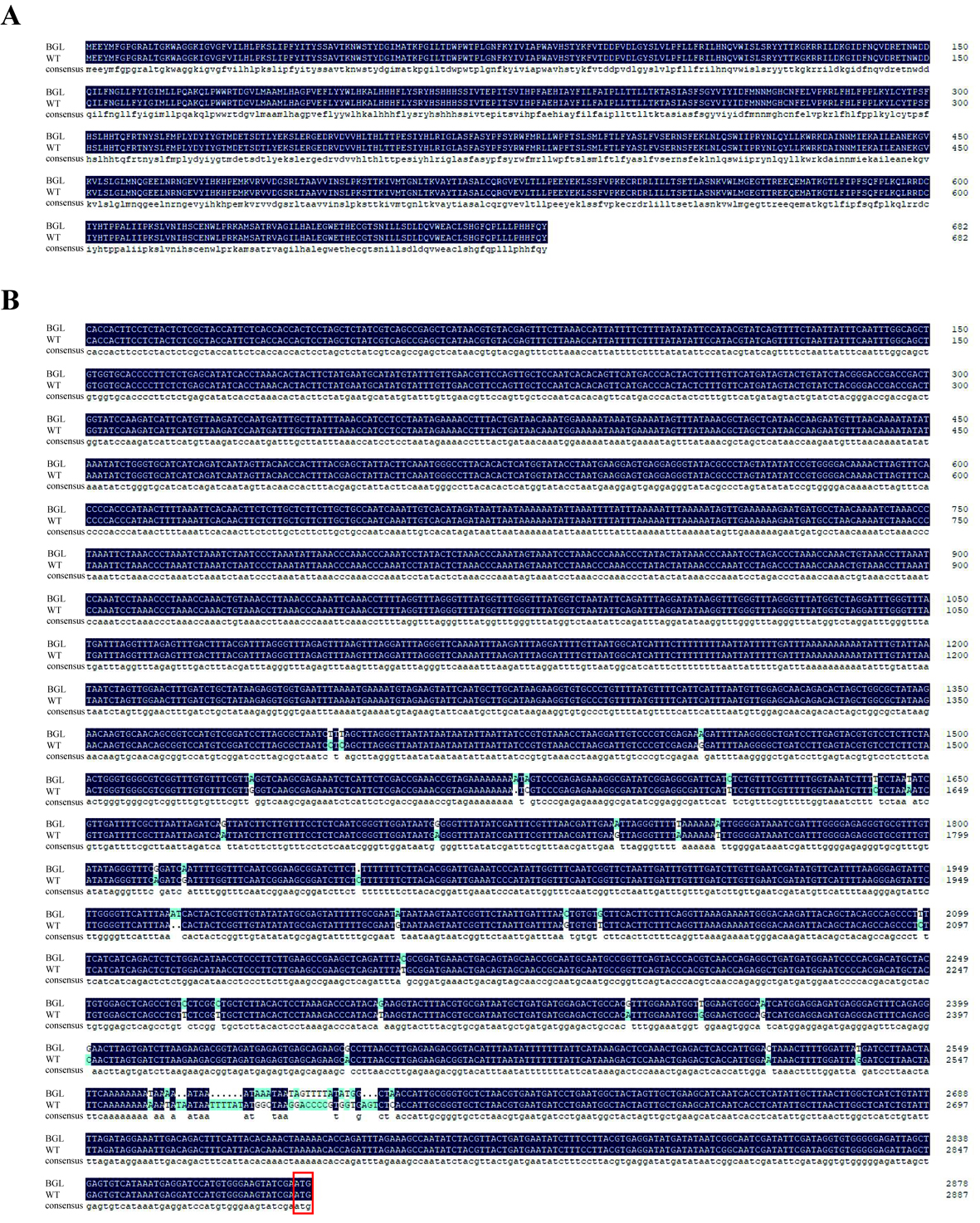

Supplement: Supplementary Figure 2 — Sequence alignment analysis of candidate gene BoCER1.C8. (A) Amino acid sequence alignment diagram of coding region between the two parents. (B) Promoter sequence alignment between the two parents. [file Image2.jpeg]
